# Native Fish Inclusion Promotes Nutrient Retention and Productivity in a Biofloc-Based Aquaponic System

**DOI:** 10.3390/ani16091404

**Published:** 2026-05-03

**Authors:** Adolfo Jatobá, Bruno Corrêa da Silva, Felipe Boéchat Vieira, Marco Shizuo Owatari, Leonardo Alexander Krause, Amanda Dartora, Maísa de Lima Lasala, Keren Fagundes Morais, Jaqueline I. A. de Andrade

**Affiliations:** 1 Aquaculture Laboratory, Catarinense Federal Institute, Campus Araquari (IFC), BR-280 Km 27, Araquari 89245-000, SC, Brazil; 2 Empresa de Pesquisa Agropecuária e Extensão Rural de Santa Catarina (EPAGRI), Rodovia Antônio Heil, 5 Itaipava, Itajaí 88318-112, SC, Brazil; 3 Laboratório de Camarões Marinhos, Departamento de Aquicultura, Universidade Federal de Santa Catarina, Rua Dos Coroas, 503, Barra da Lagoa, Florianópolis 88061-600, SC, Brazil

**Keywords:** *Astyanax bimaculatus*, biofloc technology, integrated multitrophic aquaculture, nutrient retention, sustainability

## Abstract

The integration of multiple species has been proposed as a strategy to improve resource use efficiency in intensive aquaculture systems. This study evaluated the inclusion of a native fish species, yellowtail lambari (*Astyanax bimaculatus*), in a biofloc-based aquaponic system co-cultivating Nile tilapia (*Oreochromis niloticus*) and lettuce (*Lactuca sativa* var. *capitata*). The experiment was conducted over 35 days using eight experimental units with two treatments (with and without lambari) and four replicates. Water quality, zootechnical performance, lettuce growth, hematological parameters of tilapia, and nitrogen and phosphorus retention were assessed. The presence of lambari was associated with lower total ammonia nitrogen, toxic ammonia, and total suspended solids, particularly during the final stage of the experimental period (*p* < 0.05), as well as reduced pH and alkalinity, likely reflecting increased microbial activity. Lettuce cultivated in the lambari treatment showed higher final weight, leaf height, and total biomass (*p* < 0.05), resulting in increased system productivity. No significant differences were observed in growth performance or hematological parameters of Nile tilapia (*p* > 0.05). In addition, nitrogen and phosphorus retention at the system level were higher in the lambari treatment (*p* < 0.05), although no differences were detected when fish and plants were evaluated separately. These results indicate that the inclusion of a native fish species can influence nutrient retention and productivity in biofloc-based aquaponic systems without compromising the performance of the primary cultured species.

## Simple Summary

Aquaculture is growing rapidly as a way to produce food, but it often faces challenges such as water pollution and inefficient use of nutrients. One promising solution is to combine different species in the same system so that waste from one organism can be used by another. In this study, we evaluated whether adding a small native fish (lambari) to a system already producing tilapia and lettuce could improve overall performance. The results showed that including lambari was associated with maintaining stable water quality by reducing harmful substances and suspended particles. In addition, lettuce plants grew better, producing more biomass and larger leaves. Importantly, the growth and health of tilapia were not negatively affected. The system with lambari also retained more nutrients, meaning less waste and better use of available resources. These findings suggest that adding native fish species can make aquaculture systems more efficient and sustainable. This approach may help farmers produce more food while reducing environmental impacts, contributing to more responsible and sustainable food production systems.

## 1. Introduction

Aquaculture is one of the fastest-growing food production sectors worldwide and plays an important role in global food security by supplying animal protein through a wide diversity of cultivated species and production systems [1]. However, the intensification of aquaculture has been associated with environmental challenges, particularly nutrient accumulation, water quality deterioration, and increased nitrogen and phosphorus discharge into aquatic ecosystems [2,3]. In response to these challenges, production systems based on the circular bioeconomy have emerged as promising strategies to achieve these objectives, as they prioritize productivity while also focusing on environmental sustainability and efficient resource use [4], with integrated multitrophic aquaculture (IMTA) playing a key role in this approach.

IMTA systems have gained attention as a means to improve nutrient use efficiency by coupling organisms occupying different trophic niches, allowing wastes generated by one component to be assimilated by others [2,5]. Within this context, biofloc technology (BFT) and aquaponics have emerged as distinct but potentially complementary approaches. While BFT focuses on microbial nutrient assimilation within intensive fish systems, aquaponics integrates fish and plant production through nutrient reuse. The combination of these approaches has led to biofloc-based aquaponic systems, which differ from classical IMTA by operating in recirculating freshwater environments with fewer clearly separated trophic levels [6,7]. Aquaponics integrates fish culture with hydroponic plant production, by utilizing nutrients derived from fish metabolism to support plant growth while simultaneously to water quality improvement [8,9]. The combination of these approaches has led to the development of biofloc-based aquaponic systems with increased potential for water reuse and nutrient recovery [9]. Recent efforts have introduced the concept of “FLOCponics”, an integration of biofloc technology with soilless plant production that aims to combine the nutrient cycling benefits of bioflocs with hydroponic cultivation. According to Pinho et al. [10], this approach remains in its early research stage, with still limited experimental standardization and inconsistent results reported for animal and plant yields, but highlights its potential to enhance water and nutrient use efficiency in integrated systems.

Species diversification is recognized as a strategy to enhance the sustainable development of aquaculture [11] and represents an additional opportunity to enhance the performance of integrated biofloc systems. Studies applying IMTA concepts within BFT environments have demonstrated that multitrophic integration can increase total biomass production and improve nutrient dynamics without increasing water consumption or system area. For example, Poli et al. [12] reported higher productivity and improved nitrogen management in a biofloc-based IMTA system integrating shrimp, tilapia, and halophytic plants. Similarly, Borges et al. [13] found benefits in integrating the farming of *Penaeus vannamei* and mullet (*Mugil liza*), resulting in a significant decrease in sludge and *Vibrio* spp. concentrations in the system when mullet was co-cultured with shrimp. However, studies integrating multiple biological components in freshwater biofloc-based systems remain limited, particularly those involving native fish species.

The identification of suitable fish species remains a key challenge for the optimization of freshwater biofloc-based multitrophic systems. Small native Brazilian fish species with rapid growth and short production cycles have been suggested as potential candidates for integration [14], although their functional roles within such systems are still poorly documented. In this context, the yellowtail lambari (*Astyanax bimaculatus*), a Neotropical species widely distributed in South America, has been proposed as a promising option due to its fast growth, early sexual maturation, and tolerance to intensive rearing conditions, which warrants further experimental evaluation [15,16]. Its omnivorous feeding behavior and tolerance to variable water quality conditions suggest a strong potential for its application in biofloc-based systems, including the consumption of particulate organic matter and microbial aggregates, which may contribute to the utilization of particulate organic matter and influence nutrient dynamics [6,15].

Despite these favorable characteristics, knowledge about the inclusion of lambari in integrated biofloc-based aquaponic systems is still scarce. In particular, the effects on water quality, growth performance, hematological parameters, and nutrient retention have not been sufficiently investigated [9,12]. Therefore, this study aimed to evaluate the inclusion of yellowtail lambari (*A. bimaculatus*) in a biofloc-based aquaponic system integrating Nile tilapia (*Oreochromis niloticus*) and lettuce (*Lactuca sativa* var. *capitata*), and to assess its effects on water quality, nitrogen and phosphorus retention, fish and plant performance, and the hematological profile of Nile tilapia.

## 2. Materials and Methods

Experimental units were randomly assigned to treatments. All units were maintained under identical environmental conditions, including greenhouse environment, light exposure, water circulation, aeration, and temperature control. The study was approved by the Ethics Committee on the Use of Animals (CEUA) of the Federal Institute of Santa Catarina—Araquari Campus (protocol number 372/2021). All procedures were conducted at the Aquaculture Laboratory of the Federal Institute of Santa Catarina, located in the municipality of Araquari, Santa Catarina, Brazil (26.3949° S; 48.7383° W). The experimental period (35 days) was defined based on previous studies in biofloc-based aquaponic system, as well as the short production cycle of lettuce, aiming to evaluate short-term system responses under controlled conditions [17].

### 2.1. Biological Material

Two fish species were used in the experiment: 160 Nile tilapia (*Oreochromis niloticus*) with an average weight of 95.44 ± 4.89 g, and 240 juveniles of yellowtail lambari (*Astyanax bimaculatus*) with an average weight of 1.01 ± 0.05 g, all originating from the Aquaculture Laboratory. Additionally, 96 lettuce seedlings (*Lactuca sativa* var. capitata), with an initial mean fresh weight of 6.4 ± 0.5 g, were used in the system.

### 2.2. Experimental Design and Management

The mature biofloc water used as inoculum was obtained from a previously stabilized biofloc-based system maintained after the completion of an earlier experimental trial, following the management procedures described by Martins et al. [17]. This system was continuously operated under intensive conditions with carbon supplementation to sustain an active microbial community and stable biofloc formation, ensuring high microbial biomass and nutrient recycling capacity. The initial water (mature biofloc) presented the following characteristics: total suspended solids of 747.65 mg L^−1^, total ammonia nitrogen (TAN) of 0.24 mg L^−1^, nitrite of 0.33 mg L^−1^, nitrate of 142.26 mg L^−1^, orthophosphate of 6.41 mg L^−1^, alkalinity of 160.40 mg L^−1^, pH of 7.35, water temperature of 27.94 °C, and dissolved oxygen of 6.89 mg L^−1^.

The multitrophic system was installed inside a 96-m^2^ greenhouse and consisted of eight experimental units. Each unit comprised a circular polyethylene tank with a working volume of 370 L (culture tank), a clarifier culture tank (secondary circular tank of 80 L), and a support structure holding four NFT (Nutrient Film Technique) channels, each 1 m long and set at a 7° slope.

Water recirculation was continuous. Water was first pumped from the culture tank to the clarifier using a submersible pump (Sarlo Better SB1000C; 5 L min^−1^, São Paulo, Brazil), after which it flowed through the NFT channels and returned to the culture tank by gravity. The culture tanks were aerated using circular micro-perforated hoses (AeroTube, 40 cm diameter, Brazil), whereas the clarifiers received aeration via 15 cm bar-type airstones connected to silicone airlines. Water temperature in the culture tanks was maintained between 25 and 26 °C using submersible thermostat heaters (500 W). Each experimental unit received twenty Nile tilapia. NFT channels were stocked with twelve seedlings of butterhead lettuce. Eight units were randomly assigned to two treatments with four replicates each. In the filter-feeder treatment, the 80 L tanks contained thirty *A. bimaculatus* acting as biological filtrators, whereas the control units had no fish in the smaller tank.

Tilapia were fed twice daily (08:00 and 16:00) with a commercial diet (presence, 4–6 mm; 32% crude protein) at 2.8% of biomass. Lambari were not provided with exogenous feed throughout the experimental period, aiming to stimulate the consumption of biofloc particles and suspended organic matter naturally present in the system. Sodium bicarbonate was supplied daily at 10% of the total feed offered to maintain the alkalinity levels. Water lost to evaporation was replenished as needed. Biometric measurements were performed weekly for Nile tilapia to adjust feeding rates, while lambari were measured only at the end of the experimental period.

### 2.3. Water Quality Assays

Temperature and dissolved oxygen were measured daily using oximeter (YSI Pro20, Yellow Springs, OH, USA), and floc volume were quantified with an Imhoff cone with a 30 min sedimentation period, following Avnimelech et al. [6]. Weekly analyses included alkalinity, pH (Mylabor PA 210A pH meter, São Paulo, Brazil), total ammonia nitrogen (TAN), unionized ammonia (NH_3_), nitrite (NO_2_), nitrate (NO_3_), and orthophosphate, all determined with an AT-100P photocolorimeter (ALFAKIT^®^, Florianópolis, Brazil). Total Suspended Solids (TSS) were assessed following the procedures recommended by APHA [18].

### 2.4. Animal and Vegetable Performance

After 35 days, vegetable yield was evaluated by measuring plant survival, growth, fresh biomass, and leaf number. Leaf count and individual fresh weight were recorded for each lettuce plant, along with the number of surviving individuals. For the fish (tilapia and lambari), performance indicators such as feed conversion ratio, survival, specific growth rate, yield, weekly growth, and final mean weight were calculated following the procedures described by Jatobá et al. [15]. The final combined biomass of fish and vegetables was also determined for each experimental unit.

### 2.5. Nutrient Retention

Before and after the experimental period, samples of fish, vegetables, and feed were collected from each experimental unit to determine nitrogen and phosphorus retention, following the procedures described by AOAC [19]. Retention (%) was estimated using the following formula:

Retention (%) = [(final biomass × final nutrient content (N or P)) − (starting biomass × initial nutrient content (N or P))]/nutrient input (N or P) × 100.

Evaluation of nitrogen and phosphorus retention began with the collection of 0.1 kg of Nile tilapia fry and 0.05 kg of each vegetable species at the moment the experimental units were stocked. At the end of the trial, new samples of the same mass were collected from each unit. All analyses were performed at Biohall Pesquisa e Inovação, a biotechnology company located in Itajaí, Santa Catarina, Brazil.

Fish samples were stored frozen, while vegetable samples were maintained under refrigeration at 5 °C until processing. Total nitrogen was quantified using the Kjeldahl method, in which a known amount of sample is digested with concentrated sulfuric acid (H_2_SO_4_) and specific catalysts to convert organic nitrogen to ammonium (NH_4_^+^). The digestate is then neutralized with sodium hydroxide (NaOH), releasing ammonia (NH_3_), which is distilled and trapped in a standard acid solution. Quantification is achieved by titration, allowing determination of total nitrogen content.

Available phosphorus—corresponding to the biologically accessible fraction—was determined by forming the blue phosphomolybdate complex after reduction with ascorbic acid, followed by spectrophotometric measurement. This procedure is based on dissolving phosphate minerals and displacing phosphorus bound to solid surfaces through competitive anions.

### 2.6. Hematological Analysis

At the end of the fifth experimental week, five tilapia from each experimental unit were anesthetized with eugenol (50 mg L^−1^), and blood was collected by caudal venipuncture using syringes containing 10% EDTA as an anticoagulant. For hematological examinations, duplicate blood smears were prepared and stained using the May–Grünwald, Giemsa, and Wright protocol [19] to determine total and differential leukocyte counts, using the formula bellow:

Total leukocytes = (leukocyte number × erytrocyte number)/2000 counted erythrocytes on the smears

Hematocrit values and total erythrocyte counts were obtained according to the methods of Ranzani-Paiva et al. [20]. Hematimetric indices—mean corpuscular volume (MCV), mean corpuscular hemoglobin (MCH), and mean corpuscular hemoglobin concentration (MCHC)—were subsequently calculated.

### 2.7. Statistical Analysis

The data were subjected to the Kolmogorov–Smirnov test to verify normality and to the Levene test to assess homoscedasticity. Zootechnical, phytotechnical, nutrient retention, and water quality variables met the assumptions of normality and homogeneity of variances and were therefore analyzed using Student’s *t*-test for comparison of means. A significance level of 5% was adopted for all analyses, following the procedures described by Zar [21].

Water quality variables were analyzed at each sampling time to compare treatments. However, as these data have a longitudinal structure, the results should be interpreted with caution due to the potential for increased type I error associated with multiple comparisons across time.

## 3. Results and Discussion

### 3.1. Water Quality

**Figure 1:**
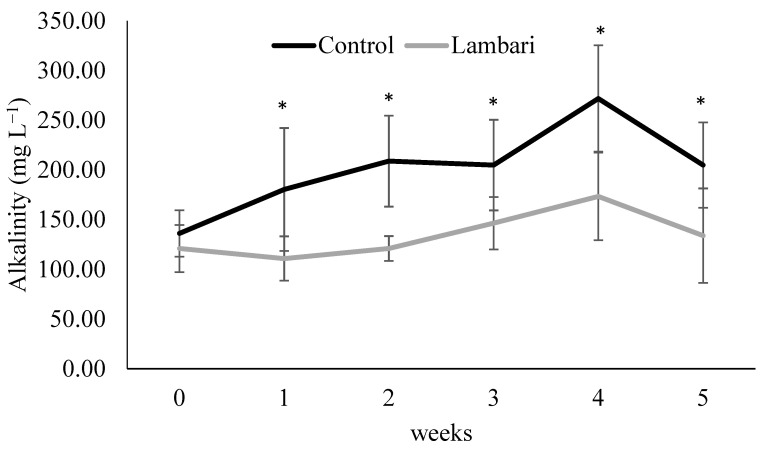
Weekly variation in alkalinity (mean ± standard deviation) in the biofloc-based IMTA system with and without yellowtail lambari (*Astyanax bimaculatus*). * Indicates statistically significant differences between groups (*p* < 0.05, *t*-test).

**Figure 2:**
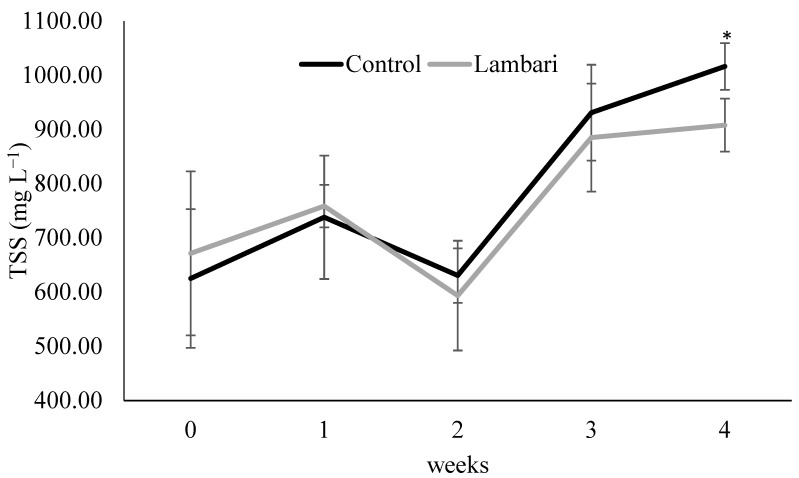
Weekly variation in total suspended solids (TSS) (mean ± standard deviation) in the biofloc-based IMTA system with and without yellowtail lambari (*Astyanax bimaculatus*). * Indicates statistically significant differences between groups (*p* < 0.05, *t*-test).

The inclusion of yellowtail lambari in the biofloc-based aquaponic system was associated with differences in key water quality parameters, particularly during the final stage of the experimental period. Lower alkalinity, total ammonia nitrogen (TAN), toxic ammonia (NH_3_), and total suspended solids (TSS) were observed in the lambari group, with statistically significant differences between treatments recorded in the final week (Figure 1 and Figure 2, Table 1).

In biofloc-based systems, alkalinity is closely linked to microbial processes, particularly nitrification, which consumes alkalinity during ammonia oxidation. Thus, the observed reduction in alkalinity may reflect increased microbial activity and associated buffering demand, rather than representing an isolated improvement in water quality. Overall, these results suggest that species diversification influenced both the chemical and particulate dynamics of the system, particularly under the conditions observed in the later stages of the experimental period.

**Figure 3:**
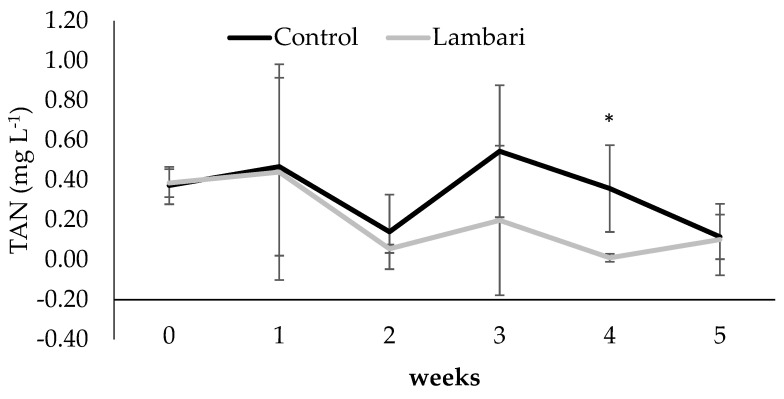
Weekly variation in total ammonia (mean ± standard deviation) in the biofloc-based IMTA system with and without yellowtail lambari (*Astyanax bimaculatus*). * Indicates statistically significant differences between groups (*p* < 0.05, *t*-test).

**Figure 4:**
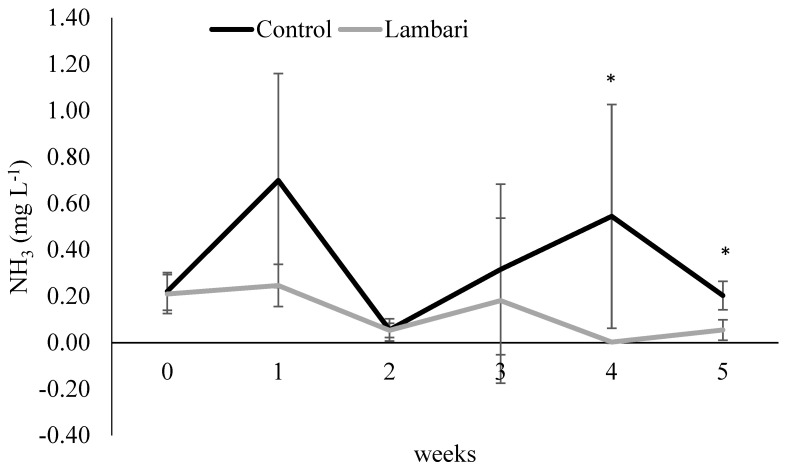
Weekly variation in NH_3_ (mean ± standard deviation) in the biofloc-based IMTA system with and without yellowtail lambari (*Astyanax bimaculatus*). * Indicates statistically significant differences between groups (*p* < 0.05, *t*-test).

Reductions in TAN (Figure 3) and NH_3_ (Figure 4) were especially evident during weeks 4 and 5. Although significant differences were detected at specific time points, particularly in the final weeks, these results should be interpreted as indicative of emerging trends rather than consistent effects over the entire experimental period.

In biofloc-based aquaculture, nitrogen dynamics are largely influenced by microbial assimilation processes stimulated by carbon supplementation, in which heterotrophic bacteria incorporate dissolved inorganic nitrogen into microbial biomass, as well as by nitrification pathways that contribute to alkalinity consumption through ammonia oxidation and proton release [6,22].

The lower TAN and NH_3_ concentrations observed in the lambari group may therefore reflect changes in these microbial processes and could be associated with higher system-level nitrogen retention (Table 2) and indirect effects on microbial activity, as previously suggested for diversified and multitrophic biofloc systems [7,12]. Similarly, the reduction in total suspended solids may reflect changes in particulate dynamics, potentially influenced by interactions between fish and suspended organic matter.

However, these mechanisms were not directly assessed in the present study and should therefore be interpreted as plausible explanations rather than confirmed processes. Overall, these results suggest that the integration of Nile tilapia, yellowtail lambari, and lettuce influenced both chemical and particulate dynamics within the system.

**Figure 5:**
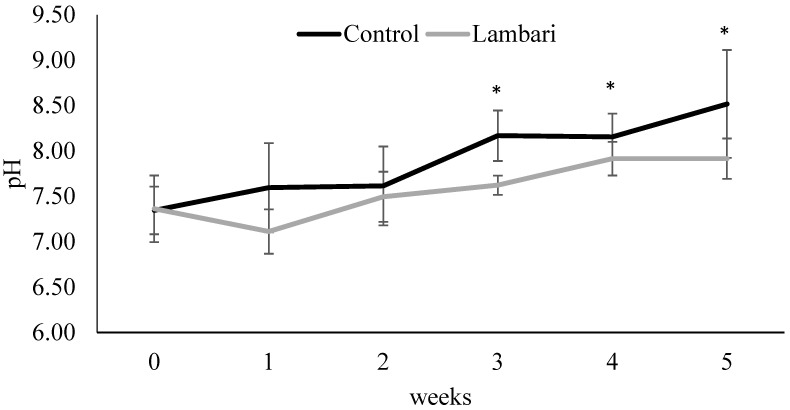
Weekly variation in pH (mean ± standard deviation) in the biofloc-based IMTA system with and without yellowtail lambari (*Astyanax bimaculatus*). * Indicates statistically significant differences between groups (*p* < 0.05, *t*-test).

Changes in pH dynamics further support the potential functional role of lambari in the system. Although pH values remained relatively high throughout the experiment (>7), a common characteristic of biofloc systems due to alkalinity management and intense microbial activity, significantly lower pH values were recorded in the lambari group during weeks 3 to 5 (Figure 5). This reduction in pH, together with the observed decrease in alkalinity, may be associated with increased microbial activity and nitrification processes, which consume alkalinity and release hydrogen ions into the system. Elevated pH levels are known to increase the proportion of toxic ammonia and may impose suboptimal conditions for both fish and leafy vegetables in integrated systems [3,6,8]. Therefore, the moderate pH reduction observed with lambari inclusion represents a potentially beneficial adjustment, bringing values closer to those considered suitable for Nile tilapia and lettuce cultivation, without negatively affecting animal performance.

Total suspended solids were significantly lower in the lambari group at the end of the experimental period. Although no consistent differences were observed throughout the entire experimental period, this result suggests a possible late-stage effect on particulate matter dynamics. In biofloc-based systems, suspended solids are primarily composed of organic particles and microbial aggregates, and their concentration is influenced by biological and physicochemical processes.

The observed reduction may be associated with the presence of lambari, potentially through interactions with suspended particles or indirect effects on floc structure and microbial aggregation. Small omnivorous fish species have been reported to ingest fine particulate material and associated microorganisms [6,12], which could contribute to changes in particulate dynamics. However, this mechanism was not directly assessed in the present study and should therefore be interpreted as a plausible explanation rather than a confirmed process.

In this context, the biological characteristics of lambari, including its feeding behavior and tolerance to suspended solids, may have contributed to the observed pattern, as also suggested by studies on its performance in intensive systems [23].

Overall, the improvements in water quality observed in the lambari group indicate that the inclusion of this Brazilian native species contributed to a more stable and chemically favorable biofloc-based aquaponic system. These findings reinforce the potential of species diversification as a practical strategy to enhance nutrient retention, mitigate nitrogen accumulation, and optimize water quality in integrated freshwater biofloc systems, while maintaining productive performance of the primary cultured species.

### 3.2. System Productivity and Biological Performance

In the lambari group, lettuce exhibited significantly higher final mean weight, leaf height, and biomass, resulting in increased overall system productivity. In contrast, no significant differences in Nile tilapia performance were observed between groups, regardless of the presence of lambari (Table 2).

In this context, lambari can be considered a complementary component within the integrated system, potentially contributing to overall system performance through its incorporation as additional biomass and possible interactions with suspended particulate matter. Although its omnivorous feeding behavior suggests the capacity to utilize fine organic particles and associated microorganisms, these processes were not directly assessed in the present study. Therefore, the functional role of lambari should be interpreted based on system-level responses rather than as evidence of specific trophic or microbial interactions.

The enhanced growth performance of lettuce observed in the lambari group may be associated with increased nutrient retention within the biofloc-based aquaponic system, potentially contributing to improved nutrient availability for plant uptake. As discussed previously, the inclusion of lambari was accompanied by lower concentrations of dissolved nitrogenous compounds and reduced total suspended solids, indicating a more efficient internal processing of organic matter and nutrients. This response is consistent with studies demonstrating that yellowtail lambari exhibit satisfactory zootechnical performance and physiological adaptation under biofloc technology conditions, contributing to organic matter consumption and nutrient transformation within the system [24]. In integrated systems, such improvements in water quality and nutrient dynamics can increase nutrient availability for plants, favoring biomass accumulation and leaf development [10,25].

Hematological parameters were evaluated as indicators of the physiological condition and health status of Nile tilapia under the different experimental conditions. The inclusion of yellowtail lambari did not significantly affect the hematological parameters of Nile tilapia, which remained within expected ranges (Table 3), indicating that the integration did not compromise fish health. Importantly, the hematological values observed for Nile tilapia remained within the reference ranges reported for the species under healthy culture conditions, indicating that the inclusion of lambari did not induce physiological stress or adverse health effects [26,27].

### 3.3. Nutrient Retention

The inclusion of lambari resulted in higher nitrogen and phosphorus retention at the system level (Table 4). However, when evaluated separately for Nile tilapia and lettuce, no significant differences were observed between treatments. This pattern suggests that the higher system-level retention may be partially associated with the presence of an additional biomass compartment in the integrated treatment.

It is important to note that nutrient retention was estimated based on nutrient accumulation in harvested biomass and does not represent a complete nutrient mass balance, as environmental losses and internal transformations were not quantified. Therefore, the observed differences should be interpreted as changes in nutrient distribution within the system rather than direct evidence of improved nutrient-use efficiency.

Previous studies on biofloc-based aquaponic systems have demonstrated that enhanced microbial activity and nutrient transformation processes can directly support higher lettuce productivity compared to conventional or less integrated systems [10]. In this context, the presence of lambari may have influenced the nutrient environment through its interaction with biofloc particles and associated microbial communities, potentially contributing to nutrient retention within the system. Although the underlying mechanisms were not directly assessed, the combined responses observed in water quality and lettuce performance in the lambari group suggest that species diversification may contribute to improved nutrient retention and overall productivity in biofloc-based aquaponic systems.

In biofloc-based systems, nutrient retention is strongly influenced by the assimilation of dissolved and particulate nutrients by cultured organisms and microbial biomass. Nile tilapia primarily utilizes nutrients through formulated feed intake, whereas smaller omnivorous species may interact more directly with suspended particulate matter and microbial aggregates [6,12]. In this context, lambari may assimilate nutrients through pathways that differ from those of tilapia, potentially enhancing the utilization of biofloc particles and associated nutrients, as species-specific differences in biofloc consumption and nutrient assimilation have been reported in biofloc systems [28]. This complementary feeding behavior could improve the overall recovery of nitrogen and phosphorus retained within the system, reducing nutrient losses and increasing internal recycling efficiency.

The improved system-level nutrient retention observed with lambari inclusion reinforces the broader concept that species diversification tends to enhance nutrient recovery and internal recycling efficiency in integrated aquaculture systems. Previous studies have demonstrated that the inclusion of additional trophic components can increase nitrogen and phosphorus retention by promoting complementary nutrient assimilation pathways and reducing nutrient losses to the environment [12,17]. Rather than increasing nutrient input, the addition of a secondary fish species may favor a more complete utilization of available nutrient pools, contributing to greater system efficiency and alignment with circular economy principles [2,9]. In this context, the results of the present study highlight the potential of lambari as a functional component in biofloc-based aquaponic systems aimed at optimizing nutrient retention and sustainability.

The IMTA production model proposed in this study, which combined the cultivation of species with complementary ecosystem functions and requirements [29], improved production efficiency, opening up new aquaculture possibilities for fish belonging to the Characidae family, renowned for their diversity in the Neotropical ichthyofauna. These findings reinforce the concept that species diversification, particularly when combining organisms from different trophic levels, represents a viable strategy to improve resource use efficiency and sustainability in intensive aquaculture systems. By promoting more complete utilization of available nutrients and reducing internal losses, integrated biofloc-based systems may contribute to higher productivity and improved environmental performance without increasing external inputs.

The inclusion of yellowtail lambari (*A. bimaculatus*) in a biofloc-based aquaponic system was associated with differences in water quality parameters, particularly during the final stage of the experimental period, as well as improved phytotechnical performance of lettuce (*L. sativa* var. *capitata*), without affecting the growth or health of the primary cultured species, Nile tilapia (*O. niloticus*). In addition, higher nitrogen and phosphorus retention was observed at the system level, along with increased overall system productivity. These findings are consistent with previous studies demonstrating that biofloc-based systems enhance nutrient recycling and improve overall system productivity, particularly through more efficient nitrogen and phosphorus utilization [27,28,30].

From a broader perspective, these findings are consistent with the principles of sustainable food production and may be relevant to discussions related to the United Nations Sustainable Development Goals, particularly those associated with food security (SDG 2), responsible production (SDG 12), and aquatic resource use (SDG 14).

## 4. Conclusions

The inclusion of yellowtail lambari (*Astyanax bimaculatus*) in the biofloc-based aquaponic system influenced water quality dynamics and increased system-level nitrogen and phosphorus retention, while improving lettuce growth without affecting the performance or hematological condition of Nile tilapia (*Oreochromis niloticus*).

The higher nutrient retention may partially reflect the incorporation of nutrients into an additional biomass compartment.

These results represent short-term responses under the tested conditions and do not necessarily indicate long-term system stability or underlying mechanisms.

Overall, lambari inclusion shows potential as a complementary component to improve nutrient retention and productivity in biofloc-based aquaponic systems.

## Figures and Tables

**Table 1:** Water quality variables (mean ± standard deviation) in a biofloc-based aquaponic system integrating Nile tilapia (*Oreochromis niloticus*) and lettuce (*Lactuca sativa* var. *capitata*), with and without yellowtail lambari (*Astyanax bimaculatus*) stocked in the clarifier.

Parameter	Control	Lambari	Significance (*p*)
Dissolved oxygen (mg L^−1^)	7.38 ± 0.26	7.44 ± 0.46	0.2941
Temperature (°C)	26.0 ± 2.07	25.36 ± 0.83	0.4052
Floc volume (mL)	49.90 ± 19.88	52.86 ± 17.20	0.4149
Alkalinity (mg L^−1^)	214.30 ± 45.07 *	137.20 ± 17.51	0.0094
pH	8.11 ± 0.99	7.61 ± 0.10	0.1772
TAN (mg L^−1^)	0.33 ± 0.04 *	0.16 ± 0.16	0.0498
NH_3_ (mg L^−1^)	0.05 ± 0.02 *	0.02 ± 0.01	0.0099
Nitrite (mg L^−1^)	0.48 ± 0.43	0.88 ± 0.42	0.1137
Nitrate (mg L^−1^)	210.35 ± 33.63	202.16 ± 15.68	0.3372
Orthophosphate (mg L^−1^)	5.05 ± 0.89	5.88 ± 0.35	0.0654
Total suspended solids (mg L^−1^)	889.00 ± 31.24 *	778.60 ± 91.97	0.0317

* Indicate significant difference in the test between groups.

**Table 2:** Zootechnical and phytotechnical performance (mean ± standard deviation) in a biofloc-based aquaponic system integrating Nile tilapia (*Oreochromis niloticus*) and lettuce (*Lactuca sativa* var. *capitata*), with and without yellowtail lambari (*Astyanax bimaculatus*) stocked in the clarifier.

Parameter	Control	Lambari	Significance (*p*)
**Tilapia**			
SGR (%. day^−1^)	0.45 ± 0.02	0.45 ± 0.06	0.4797
Survival (%)	98.75 ± 4.79	100.00 ± 0.00	0.3524
Final mean weight (g)	140.11 ± 11.01	140.88 ± 7.46	0.4558
Feed conversion	2.24 ± 0.30	2.13 ± 0.20	0.2820
Yield (kg m^−3^)	6.90 ± 0.34	7.04 ± 0.31	0.2887
**Lambari**			
SGR (%. day^−1^)	-	0.73 ± 0.09	-
Survival (%)	-	72.50 ± 3.19	-
Final mean weight (g)	-	1.87 ± 0.14	-
Feed conversion	-	0.57 ± 0.20	-
Yield (kg m^−3^)	-	0.51 ± 0.05	
**Lettuce**			
Final mean weight (g)	39.47 ± 19.40	56.58 ± 19.50 *	0.0277
Root height (cm)	16.53 ± 6.25	21.66 ± 4.68	0.1187
Leaf height (cm)	20.69 ± 0.73	26.01 ± 1.93 *	0.0011
Survival (%)	100.00 ± 0.00	100.00 ± 0.00	-
Biomass (g)	463.74 ± 133.20	678.93 ± 59.02 *	0.0127
System (tilapia + lambari + lettuce)			
Yield (kg m^−3^)	7.17 ± 0.31	7.36 ± 0.22 *	0.0225

* Indicate significant difference in the test between groups.

**Table 3:** Hematological profile of Nile tilapia (*Oreochromis niloticus*) in a biofloc-based aquaponic system with lettuce (*Lactuca sativa* var. *capitata*), with and without yellowtail lambari (*Astyanax bimaculatus*) stocked in the clarifier.

Parameter	Control	Lambari	Significance (*p*)
Total and differential count			
Thrombocytes (×10^3^ µL^–1^)	15.78 ± 2.49	17.02 ± 3.18	0.3531
Total leukocytes (× 10^4^ µL^−1^)	22.30 ± 3.24	21.94 ± 2.84	0.3654
Lymphocytes (×10^3^ µL^–1^)	20.35 ± 2.87	19.64 ± 1.79	0.4004
Monocytes (×10^3^ µL^–1^)	1.41 ± 0.55	1.56 ± 0.49	0.4175
Neutrophils (×10^3^ µL^–1^)	0.54 ± 0.32	0.39 ± 0.30	0.4918
Hematimetric indexes			
Erythrocytes (×10^6^ µL^–1^)	1.65 ± 0.29	1.58 ± 0.33	0.5486
Hematocrit (%)	25.72 ± 1.11	26.03 ± 0.67	0.6462
Hemoglobin (g dL^–1^)	0.22 ± 0.01	0.23 ± 0.01	0.3805
Total plasma proteins (mg L^–1^)	1041.06 ± 2.13	1039.97 ± 0.54	0.3591
MCV (fL)	2.44 ± 1.93	2.69 ± 2.41	0.4782
MCH (pg)	3.75 ± 0.70	4.05 ± 0.62	0.3981
MCHC (g dL^–1^)	2.15 ± 0.41	2.01 ± 0.49	0.4422

Mean corpuscular volume (MCV); mean corpuscular hemoglobin (MCH); mean corpuscular hemoglobin concentration (MCHC).

**Table 4:** Nitrogen and phosphorus retention (mean ± standard deviation) in a biofloc-based aquaponic system integrating Nile tilapia (*Oreochromis niloticus*) and lettuce (*Lactuca sativa* var. *capitata*), with and without yellowtail lambari (*Astyanax bimaculatus*) stocked in the clarifier.

Parameter	Control	Lambari	Significance (*p*)
Tilapia			
N	22.49 ± 1.09	22.25 ± 0.56	0.7027
P	21.89 ± 0.78	22.56 ± 0.45	0.1894
Lambari			
N	-	0.34 ± 0.19	-
P	-	4.57 ± 0.95	-
Lettuce			
N	3.48 ± 0.61	4.35 ± 1.17	0.2351
P	0.73 ± 0.38	0.77 ± 0.29	0.8476
System (tilapia + lambari + lettuce)			
N	25.35 ± 0.37	27.31 ± 1.08 *	0.0179
P	20.12 ± 3.86	27.90 ± 1.02 *	0.0080

* Indicate significant difference in the test between groups.

## Data Availability

The data presented in this study are available on request from the corresponding author.

## Abbreviations

The following abbreviations are used in this manuscript:

IMTA	Integrated Multitrophic Aquaculture
BFT	Biofloc Technology
TAN	Total Ammonia Nitrogen
NFT	Nutrient Film Technique
NH_3_	Unionized Ammonia
NO_2_	Nitrite
NO_3_	Nitrate
TSS	Total Suspended Solids
MCV	Mean Corpuscular Volume
MCH	Mean Corpuscular Hemoglobin
MCHC	Mean Corpuscular Hemoglobin Concentration
